# THOC2 and THOC5 Regulate Stemness and Radioresistance in Triple‐Negative Breast Cancer

**DOI:** 10.1002/advs.202102658

**Published:** 2021-10-27

**Authors:** Xupeng Bai, Jie Ni, Julia Beretov, Shanping Wang, Xingli Dong, Peter Graham, Yong Li

**Affiliations:** ^1^ St George and Sutherland Clinical School Faculty of Medicine UNSW Sydney Kensington NSW 2052 Australia; ^2^ Cancer Care Centre St George Hospital Kogarah NSW 2217 Australia; ^3^ Anatomical Pathology NSW Health Pathology St George Hospital Kogarah NSW 2217 Australia; ^4^ Institute of Biomedical and Pharmaceutical Sciences Guangdong University of Technology Guangzhou 510006 China; ^5^ Department of Biopharmaceutical Sciences College of Pharmacy Harbin Medical University Harbin 150081 China; ^6^ School of Basic Medicine Zhengzhou University Zhengzhou 450001 China

**Keywords:** cancer stem cells, NANOG, radioresistance, SOX2, THO2, THOC5, TNBC

## Abstract

Triple‐negative breast cancer (TNBC) is the most aggressive subtype of breast cancer. Radioresistance and stemness are substantial obstacles to TNBC treatment. The THO complex (THOC) is a subunit of the TRanscription–EXport complex that functions in the coupling of transcription to nascent RNA splicing, elongation, and export. However, its role in regulating TNBC therapeutic resistance is not reported yet. In this study, the authors demonstrate that cancer stem cells are enriched in radioresistant TNBC cells and describe the role of the THOC in regulating TNBC radioresistance and stemness. The authors find that THOC2 and THOC5 are upregulated in radioresistant TNBC cells and associated with a poor prognosis in TNBC patients. Further investigation reveals that THOC2 promotes the stem‐like properties and radioresistance of TNBC cells in a THOC5‐dependent manner by facilitating the release of sex‐determining region Y (SRY)‐box transcription factor 2 (SOX2) and homeobox transcription factor (NANOG) transcripts from the nucleus. Silencing THOC2 or THOC5 expression decreases the protein expression of SOX2 and NANOG, depletes the stem‐like properties, and causes radiosensitization in these TNBC cells. Moreover, THOC2 or THOC5 depletion blocks the xenograft tumorigenesis and growth of radioresistant TNBC in vivo. These findings uncover the novel correlations of THOC with TNBC stemness and therapeutic resistance, proposing alternative therapeutic strategies against relapsed TNBC.

## Graphical abstract

This study reveals a critical role of the THO complex (THOC) in cancer stemness enhancement by regulating the transnuclear export of pluripotency transcripts, which is a novel mechanism contributing to triple‐negative breast cancer (TNBC) radioresistance. These findings propose THOC2 and THOC5 as novel therapeutic targets for TNBC by eliminating the stem‐like properties of the tumor.

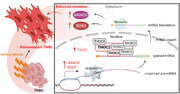

## 1 Introduction

Triple‐negative breast cancer (TNBC) has a higher risk of recurrence and death than other breast cancer (BC) subtypes though accounts for only 10–30% of all BC cases.^[^ ^1^ ^]^ Due to the absence of estrogen receptor, human epithermal growth factor receptor 2, and progesterone receptor, it is unable to benefit from traditional BC targeted therapies, and thus finding out alternative targets for TNBC treatment has always been a prominent topic in BC research.

Radiotherapy (RT), as an option for locoregional cancer treatment, is increasingly utilized in the postoperative management of TNBC,^[^ ^2^ ^]^ particularly with the recognition of the effectiveness of breast‐conserving surgery in TNBC treatment.^[^ ^3^ ^]^ Postoperative RT could reduce the 10‐year recurrence rate of TNBC from 35.0% to 19.3% and significantly improved the 15‐year survival rate.^[^ ^4^ ^]^ However, compared with other BC subtypes, TNBC is more resistant to ionizing radiation (IR), which often leads to undetected metastatic dissemination and relapse after RT.^[^ ^5^ ^]^ Recurrent or advanced TNBCs usually show poor response to subsequent therapies, and the median survival time of these patients is only approximately 18 months.^[^ ^6^ ^]^ Therefore, elucidating the mechanisms underlying TNBC radioresistance and targeting the vulnerability are of great importance for TNBC treatment.

Cancer stem cells (CSCs) are functionally defined as cancer cells with the capacity to self‐renew and recapitulate tumor heterogeneity. Mounting evidence indicates that conventional therapies for BC, including chemotherapy,^[^ ^7^ ^]^ RT,^[^ ^8^ ^]^ and hormone therapy,^[^ ^9^ ^]^ often increase the proportion of CSCs in the tumor. In many cases, these self‐renewal CSCs are suggested to mediate the therapeutic resistance and metastatic spread by enhanced DNA repair and epithelial–mesenchymal transition (EMT), respectively, resulting in treatment failure and disease recurrence.^[^ ^10^ ^]^ Compared with other BC subtypes, CSCs with CD44^+^CD24^−/low^ expression signatures and/or high aldehyde dehydrogenase (ALDH) activity are more enriched in TNBC tissues and cell lines,^[^ ^11^ ^]^ and the pathways associated with stemness, such as the Janus kinase 2 (JAK2)/signal transducer and activator of transcription 3 (STAT3) signaling,^[^ ^12^ ^]^ tyrosine‐protein kinase Src signaling,^[^ ^13^ ^]^ Hedgehog signaling,^[^ ^14^ ^]^ and Wnt/*β*‐catenin signaling,^[^ ^15^ ^]^ are more activated in TNBC tumors. Given that the treatment of TNBC still relies on conventional options, targeting the stemness and eradicating intratumoral CSCs may help improve TNBC treatment outcomes.

The THO complex (THOC) is a subunit of the TRanscription–EXport (TREX) ribonucleoprotein complex that functions in the coupling of transcription to nascent RNA splicing, elongation, and export.^[^ ^16^ ^]^ In mammals, the THOC is comprised of THOC1, 2, 3, 5, 6, and 7. Notably, THOC2 acts as a THO scaffold, and THOC5 acts as an adaptor for spliced mRNA release from the nucleus. Both proteins regulate the expression of a wide range of genes and control embryonic development, especially proliferation and differentiation status.^[^ ^17^ ^]^ Wang et al.^[^ ^18^ ^]^ found that THOC2 and THOC5 could regulate the self‐renewal capacities of mouse embryonic stem cells by controlling the expression of a set of pluripotency proteins, such as estrogen‐related receptor *β* (ESRR*β*), Krüppel‐like factor 4 (KLF4), sex‐determining region Y (SRY)‐box transcription factor 2 (SOX2), and NANOG homeobox transcription factor (NANOG). Additionally, Yuan, et al. ^[^ ^19^ ^]^ found that THOC2 and THOC5 determined the differentiation phenotype of the human vascular smooth muscle cell (VSMC) by switching VSMC markers’ expression. However, the role of THOC2 and THOC5 in regulating cancer stemness is not reported yet, and the underlying mechanism remains unclear.

In this study, we show that CSCs were enriched in radioresistant TNBC cells, whilst high expression of THOC2 and THOC5 was also found in these cells and associated with a poor prognosis in TNBC patients. Further investigation reveals that THOC2 regulated the stemness and radiosensitivity of TNBC in a THOC5‐dependent manner by controlling the release of SOX2 and NANOG transcripts from the nucleus. These findings demonstrate for the first time the importance of THOC2 and THOC5 in the regulation of cancer stemness and radioresistance, both proposing novel therapeutic targets for the treatment of relapsed TNBC.

## 2 Results

### 2.1 The Enhanced Stem‐Like Property Is Found in Radioresistant TNBC Cells

**Figure 1:**
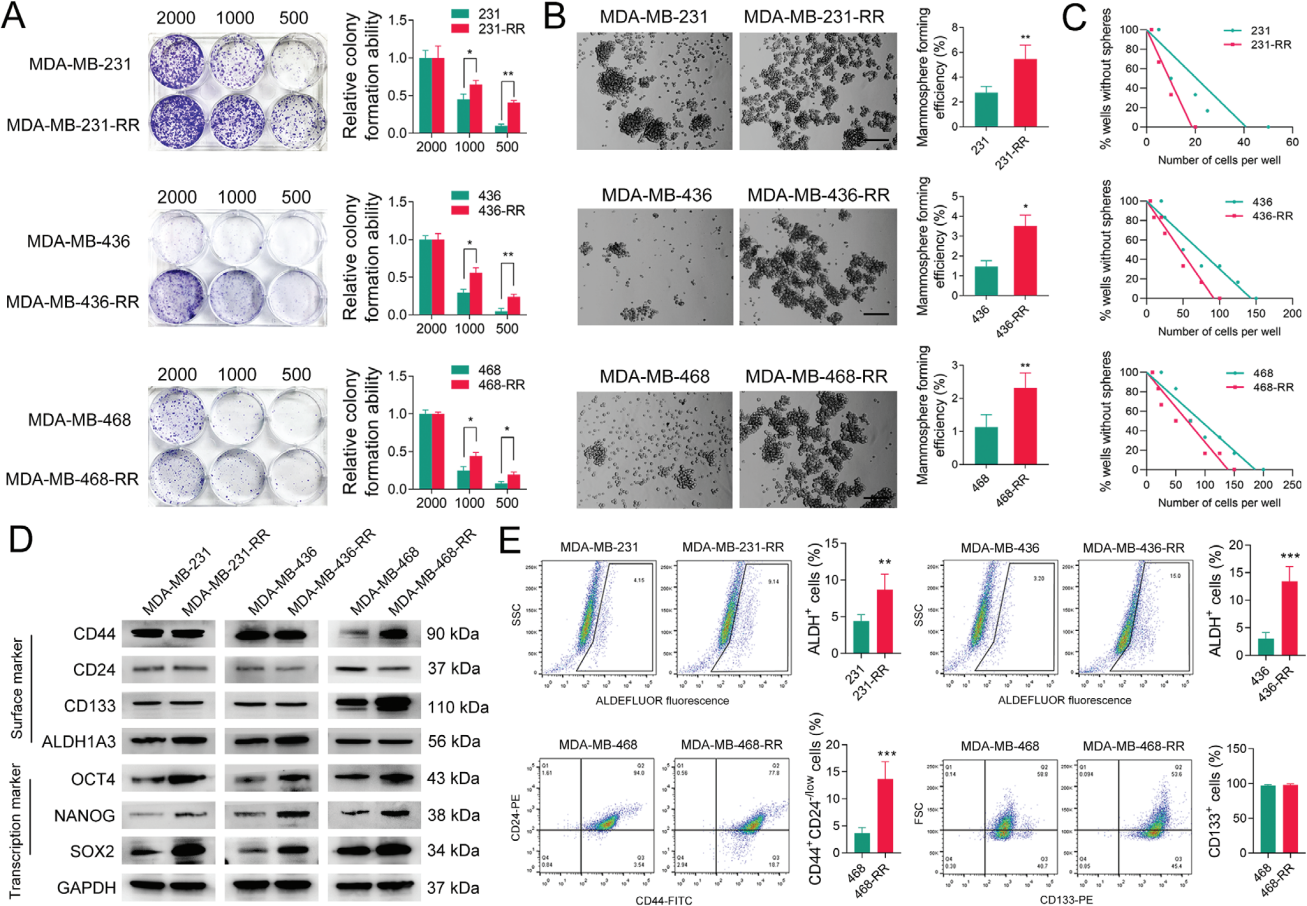
The enhanced stem‐like property is found in radioresistant TNBC cells. A) The clonogenic ability of TNBC cell lines was evaluated by colony formation assay using a limiting dilution method. Cells were seeded in the 6‐well plate at a density of 500, 1000, or 2000 cells per well and cultured for 9–14 days. B) TNBC cell lines were cultured for mammosphere formation, and the formation efficiency was calculated. Representative images are shown at 100× magnification. C) The mammosphere‐forming efficiency of TNBC cell lines was evaluated by mammosphere formation assay using a limiting dilution method, and the regression lines were generated. D) The protein expression of CD44, CD24, CD133, ALDH1A3, OCT4, NANOG, and SOX2 in TNBC cell lines was detected by WB. GAPDH was used as the loading control. E) The percentage of ALDH^+^ cells or CD44^+^CD24^−/low^ cells in radioresistant and parental TNBC cell lines was detected by flow cytometry. *P < 0.05, **P < 0.01, and ***P < 0.001 versus parental cells (*n* = 3).

We previously developed radioresistant TNBC cell lines using fractional IR and demonstrated their survival advantage upon IR‐induced cell death.^[^ ^20^ ^]^ Considering the role of CSCs in therapeutic resistance, we aim to investigate whether the developed radioresistant phenotypes are associated with the stemness of TNBC in this study. Using colony formation assays with a limiting‐dilution method, we found that radioresistant TNBC cells could form more colonies than their parental cells when the number of initially seeded cells was decreased (**Figure** **1**A). The results from mammosphere formation show that more mammospheres appeared in radioresistant TNBC cells than the parental cells (Figure 1B). Consistently, the limiting‐dilution analysis of mammosphere formation assay shows that the number of radioresistant TNBC cells required to generate a sphere is much less than the parental cells (Figure 1C), suggesting that radioresistant TNBC cells had higher mammosphere‐forming efficiency.

The CD44, CD24, CD133, and ALDH1A3 are important surface markers for CSCs in TNBC.^[^ ^21^ ^]^ Octamer‐binding transcription factor 4 (OCT4), NANOG, and SOX2 are considered central transcriptional markers in maintaining the CSC population.^[^ ^22^ ^]^ Thus, the expression of these CSC markers was also assessed to determine the stemness in radioresistant TNBC cells. As shown in Figure 1D, these markers show the characteristics of heterogeneous expression among different TNBC cell lines. The expression of ALDH1A3 was upregulated in only MDA‐MB‐231‐RR and 436‐RR cells, while the expression of CD44 and CD133 was only upregulated in MDA‐MB‐468‐RR cells (Figure 1D). Besides, the CD24 expression in MDA‐MB‐468‐RR cells was down‐regulated as compared to parentals (Figure 1D). Notably, the expression of OCT4, NANOG, and SOX2 that are crucial in supporting the pluripotent phenotype of stem cells was significantly upregulated in three radioresistant TNBC cell lines (Figure 1D). Further investigation using flow cytometry demonstrated that ALDH^+^ cells were enriched in MDA‐MB‐231‐RR and 436‐RR cells, and CD44^+^CD24^−/low^ cells in MDA‐MB‐468‐RR cells, as compared to parental cells (Figure 1E), indicating the expansion of the CSC population in radioresistant TNBC cells.

### 2.2 The Expression of THOC2 and THOC5 is Upregulated in TNBC Cells and Associated with a Worse Prognosis in Patients

**Figure 2:**
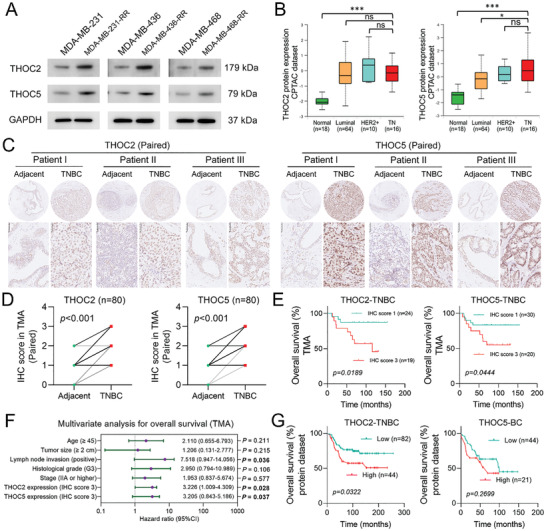
The expression of THOC2 and THOC5 is upregulated in TNBC cells and associated with a worse prognosis in patients. A) The protein expression of THOC2 and THOC5 in TNBC cell lines was detected by WB. GAPDH was used as the loading control. B) The box‐and‐whisker plots were generated using the UALCAN to describe THOC2 and THOC5 protein expression differences among human normal breast tissues and various BC subtypes. The protein expression data were obtained from the CPTAC database. C) The protein level of THOC2 and THOC5 in human TNBC tissues and paired adjacent normal breast epithelia in TMA was evaluated by IHC. Representative IHC images are displayed at 100× and 400× magnification. Brown represents target protein staining, while blue represents the nuclei. D) The protein staining of THOC2 and THOC5 by IHC between TNBC tissues and paired adjacent normal breast epithelia was scored and compared (*n* = 80). E) The association of THOC2 and THOC5 protein levels with OS in TMA was analyzed according to the IHC score. F) The hazard ratio plot (Forest plot) was generated with the results of multivariate analysis by Cox regression model for OS in TMA. G) The association of THOC2 and THOC5 protein expression with OS was analyzed using the dataset obtained from the work of Liu, et al. ^[^ ^23^ ^]a^ and Tang et al. ^[^ ^23^ ^b]^, respectively. *P < 0.05 and ***P < 0.001; ns, nonsignificant.

Both THOC2 and THOC5 play a critical role in regulating the phenotypes of stem cells.^[^ ^17^ ^]^ In this study, we found that THOC2 and THOC5 were upregulated in radioresistant TNBC cells at both mRNA and protein level (Figure S1, Supporting Information; **Figure** **2**A). Thus we next analyzed several online human cancer tissue datasets, including the Cancer Genome Atlas (TCGA) and Clinical Proteomic Tumor Analysis Consortium (CPTAC), and found that the expression of THOC2 and THOC5 was upregulated in TNBC tissues at both mRNA and protein level in comparison to normal breast tissues, and THOC5 was more specifically upregulated in human TNBC tissues (Figure S2, Supporting Information; Figure 2B). To further determine the expression of THOC2 and THOC5 in human tissues, we detected the protein level of THOC2 and THOC5 in TNBC tissue microarray (TMA) with paired adjacent normal breast epithelia using immunohistochemistry (IHC). The results from IHC show that the protein level of THOC2 and THOC5 was significantly increased in TNBC tissues, as compared to paired normal tissues (Figure 2C,D).

To address the clinical significance of THOC2 and THOC5 in TNBC, we also performed the survival analysis based on the cancer tissue IHC scoring in TMAs. The results show that patients with strong staining of THOC2 or THOC5 (IHC score = 3) in the TNBC tissues had a much lower overall survival (OS) rate than those with weak staining (IHC score = 1) (Figure 2E). Correlation analysis of THOC2 and THOC5 expression with TMA clinicopathological variables shows that high expression of THOC2 was associated with larger tumor size (*p* = 0.029) and lymph node invasion (*p* = 0.027) (Tables S1 and S2, Supporting Information). Furthermore, multivariate analysis shows that both THOC2 and THOC5 expression are the independent prognostic factors for OS (*p* = 0.028 and 0.037, respectively) (Figure 2F). Through analyzing the published databases containing patients’ survival and intratumoral proteomics data,^[^ ^23^ ^]^ we found that a higher protein expression of THOC2 in tumors was associated with a much lower OS rate in TNBC patients; THOC5 was associated with a lower OS rate in BC though not significantly (Figure 2G). Besides, analysis of the Molecular Taxonomy of Breast Cancer International Consortium (METABRIC) dataset shows that the high intratumoral expression of *THOC2* gene expression was remarkably associated with a worse prognosis in BC and TNBC patients, while *THOC5* displayed no relevance to survival (Figure S3, Supporting Information). These results indicate that the expression of THOC2 and THOC5 is associated with TNBC radioresistance and may play an essential role in TNBC progress.

### 2.3 THOC2 Knockdown Decreases the Stemness of Radioresistant TNBC Cells and Causes Radiosensitization

**Figure 3:**
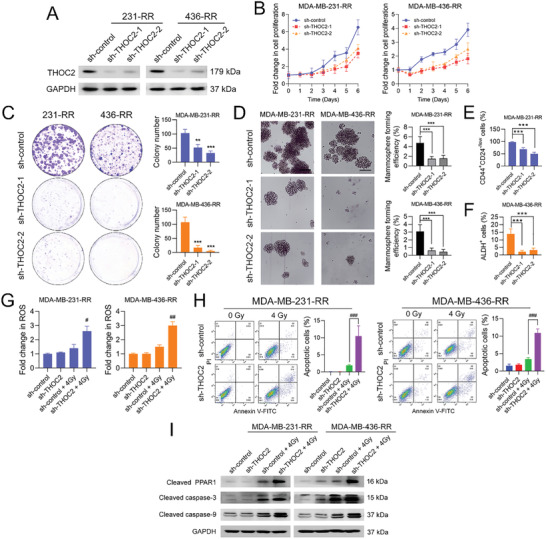
THOC2 knockdown decreases the stemness of radioresistant TNBC cells and caused radiosensitization. THOC2 was silenced in MDA‐MB‐231‐RR and 436‐RR cells using a lentiviral system with two different shRNAs. A) The protein expression of THOC2 in TNBC cell lines was detected by WB. GAPDH was used as the loading control. B) The proliferation rate of cells was determined using the proliferation assay. C) The clonogenic ability of cells was evaluated by colony formation assay. D) The stemness of TNBC cell lines was evaluated by mammosphere formation assay, and the formation efficiency was calculated. Representative images are shown at 100× magnification. E) The percentage of CD44^+^CD24^−/low^ cells in MDA‐MB‐231‐RR cells was detected using flow cytometry. F) The percentage of ALDH^+^ cells in MDA‐MB‐436‐RR cells was detected by flow cytometry. THOC2‐depleted TNBC cells were treated with 0 or 4 Gy IR. G) The intracellular ROS level was detected 24 h after IR. H) Cell apoptosis was analyzed 24 h after IR using flow cytometry, and the percentage of apoptotic cells was calculated as the percentage of cells in Q2 and Q3. I) The protein expression of cleaved PARP1, caspase‐3, and caspase‐9 was detected by WB 24 h after IR. GAPDH was used as the loading control. **P < 0.01 and ***P < 0.001 versus sh‐control group; ^#^ *P* < 0.05, ^##^ *P* < 0.01, and ^###^ *P* < 0.001 versus sh‐control + IR group (*n* = 3).

Based on the findings above, we decided to explore whether THOC2 inhibition could change the radiation response of radioresistant TNBC cells. To this end, two different short hairpin RNAs (shRNAs) that target different transcripts of THOC2 were employed in this study to reduce the expression of THOC2. Results from Western blotting (WB) show that the two THOC2‐specific shRNAs dramatically decreased the protein expression of THOC2 in MDA‐MB‐231‐RR and 436‐RR cells (**Figure** **3**A). The inhibition of THOC2 also significantly impaired the proliferation of radioresistant TNBC cells (Figure 3B), as well as their ability to form colonies (Figure 3C). Furthermore, the mammosphere formation assay shows that their sphere formation efficiency was markedly disrupted by THOC2 knockdown (Figure 3D). Further investigation from flow cytometry shows that THOC2 knockdown significantly decreased the percentage of CD44^+^CD24^−/low^ cells in MDA‐MB‐231‐RR cells and the percentage of ALDH^+^ cells in MDA‐MB‐436‐RR cells (Figure 3E,F).

To assess the effect of THOC2 inhibition on radiosensitivity, we treated radioresistant TNBC cells with 0 or 4 Gy IR after the knockdown. As depicted in Figure 3G, we observed that THOC2‐specific shRNA significantly increased IR‐induced intracellular accumulation of reactive oxygen species (ROS) in MDA‐MB‐231‐RR and 436‐RR cells. Moreover, THOC2 knockdown, combined with IR, caused more apoptotic cells in radioresistant TNBC cells than IR alone (Figure 3H). To further determine the effect of THOC2 knockdown on IR‐induced apoptosis, we also evaluated the status of mitochondrial apoptosis signaling. As shown in Figure 3I, single THOC2 shRNA did not activate the apoptosis signaling, whereas when exposed to 4 Gy IR, THOC2 shRNA significantly increased protein expressions of cleaved poly(ADP‐Ribose) polymerase 1 (PARP1), caspase‐3, and caspase‐9 in radioresistant TNBC cells, as compared to the sh‐control group. These data suggest that THOC2 inhibition can compromise the stemness of radioresistant TNBC cells and restore their sensitivity to IR. Besides, THOC2 knockdown also sensitized radioresistant TNBC cells to cisplatin and doxorubicin (Figure S4A, Supporting Information).

### 2.4 THOC2 Knockdown Decreases the Protein Expression of SOX2 and NANOG by Disrupting Their Transcript Export

**Figure 4:**
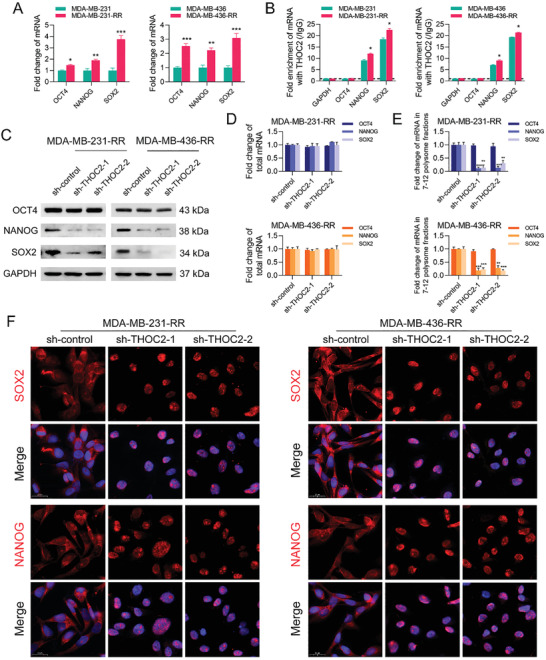
THOC2 knockdown decreases the protein expression of SOX2 and NANOG by disrupting their transcript export. A) The relative mRNA expression of OCT4, NANOG, and SOX2 in TNBC cell lines was detected by qRT‐PCR. B) The enrichment of GAPDH, OCT4, NANOG, and SOX2 mRNAs against THOC2 and IgG antibodies in TNBC cell lines was analyzed by RIP assay. THOC2 was silenced in MDA‐MB‐231‐RR and 436‐RR cells using a lentiviral system with two different shRNAs. C) The protein expression of OCT4, NANOG, and SOX2 was detected by WB. GAPDH was used as the loading control. D) The total mRNA expression of OCT4, NANOG, and SOX2 was detected by qRT‐PCR. E) The relative expression of polysome‐associated OCT4, NANOG, and SOX2 mRNAs (fractions 7–12) was detected by qRT‐PCR. F) The intracellular distribution of NANOG, SOX2, and GAPDH mRNAs was evaluated by RNA‐FISH. Representative images are shown at 630× magnification. Red represents target mRNA, while blue represents the nuclei. *P < 0.05, **P < 0.01, and ***P < 0.001 versus parental cells or sh‐control group (*n* = 3).

Consistent with Figure 1D, we observed that the mRNA expression of OCT4, NANOG, and SOX2 was upregulated in radioresistant TNBC cells (**Figure** **4**A). Given that the THOC controls the nuclear export of pluripotency gene transcripts in mouse embryonic stem cells,^[^ ^18^ ^]^ we were interested in investigating whether THOC2, as a scaffold for the THOC, plays a similar role in human TNBC. Accordingly, we first carried out RNA immunoprecipitation (RIP) assays using the antibody against THOC2 and analyzed mRNAs coimmunoprecipitated with THOC2. The results show a high binding activity of THOC2 with NANOG and SOX2 mRNAs in human TNBC cells, and this interaction was further accelerated after radioresistance; in contrast, OCT4 and GAPDH transcripts were not pulled down (Figure 4B). These data suggest that THOC2 could directly bind to NANOG and SOX2 transcripts.

To further unravel the internal association of THOC2 with pluripotency factor expression, we performed WB analysis and found that THOC2 depletion strongly decreased NANOG and SOX2 protein expression but not OCT4 in radioresistant TNBC cells (Figure 4C). Notably, whilst the total mRNA expression of NANOG and SOX2 was not perturbed by THOC2 silence (Figure 4D), their mRNA levels in 7–12 polysome fractions were dramatically decreased after THOC2 depletion (Figure 4E). To determine whether THOC2 knockdown reduces the expression of pluripotency actors by impeding the transcript releasing in TNBC, we next performed the RNA fluorescence in situ hybridization (FISH) assay and found that THOC2 knockdown caused SOX2 and NANOG transcripts to predominantly accumulate in the nuclei compared with those predominantly localized to the cytoplasm in the sh‐control group (Figure 4F). The OCT4 transcripts were not affected by THOC2 interference (Figure S5, Supporting Information). These results suggest that THOC2 may regulate TNBC cell stemness by controlling the pluripotency gene's nuclear transcript export.

### 2.5 THOC5 Is Required for THOC2‐Mediated Stemness Enhancement in Radioresistant TNBC Cells

**Figure 5:**
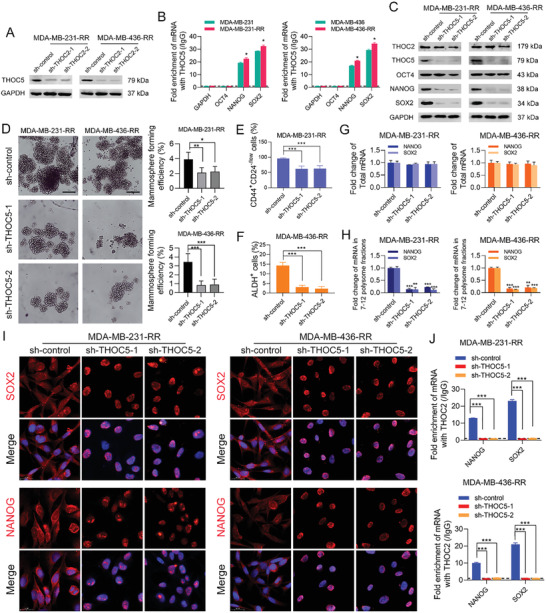
THOC5 is required for THOC2‐mediated stemness enhancement in radioresistant TNBC cells. A) THOC2 was silenced in MDA‐MB‐231‐RR and 436‐RR cells using a lentiviral system with two different shRNAs. The protein expression of THOC5 was detected by WB. GAPDH was used as the loading control. B) The enrichment of GAPDH, OCT4, NANOG, and SOX2 mRNAs against THOC5 and IgG antibodies in TNBC cell lines was analyzed by RIP assay. THOC5 was silenced in MDA‐MB‐231‐RR and 436‐RR cells using a lentiviral system with two different shRNAs. C) The protein expression of THOC2, THOC5, OCT4, NANOG, and SOX2 was detected by WB. GAPDH was used as the loading control. D) The stemness of TNBC cell lines was evaluated by mammosphere formation assay, and the formation efficiency was calculated. Representative images are shown at 100 × magnification. E) The percentage of CD44^+^CD24^−/low^ cells in MDA‐MB‐231‐RR cells was detected using flow cytometry. F) The percentage of ALDH^+^ cells in MDA‐MB‐436‐RR cells was detected using flow cytometry. G) The total mRNA expression of NANOG and SOX2 in TNBC cell lines was detected by qRT‐PCR. H) The relative expression of polysome‐associated NANOG and SOX2 mRNAs (fractions 7–12) was detected by qRT‐PCR. I) The intracellular distribution of NANOG and SOX2 mRNAs was evaluated by RNA‐FISH. Representative images are shown at 630 × magnification. Red represents target mRNA, while blue represents the nuclei. J) The enrichment of NANOG and SOX2 mRNAs against THOC2 and IgG antibodies in TNBC cell lines was analyzed by RIP assay. *P < 0.05, **P < 0.01, and ***P < 0.001 versus parental cells or sh‐control group (*n* = 3).

It has been documented that THOC5 is a specific adapter subunit of THOC for pluripotency gene transcripts,^[^ ^24^ ^]^ and in this study, we found that the protein expression of THOC5 was decreased by THOC2 knockdown in radioresistant TNBC cells (**Figure** **5**A). To corroborate the role of THOC5 in regulating TNBC stemness, we also performed RIP assays using the antibody against THOC5. The results show that NANOG and SOX2 mRNAs were co‐immunoprecipitated with THOC5, and the binding activities were further increased after radioresistance (Figure 5B). These data suggest that THOC5 could directly bind to NANOG and SOX2 transcripts. Next, we used two different shRNAs to silence THOC5 expression in MDA‐MB‐231‐RR and 436‐RR cells. Similar to THOC2 knockdown, THOC5 depletion dramatically decreased the protein expression of NANOG and SOX2 in radioresistant TNBC cells (Figure 5C). Further investigation by mammosphere formation assay shows that THOC5 silence significantly impaired the sphere formation capacities of radioresistant TNBC cells (Figure 5D). The percentage of CD44^+^CD24^−/low^ cells in MDA‐MB‐231‐RR cells and the percentage of ALDH^+^ cells in MDA‐MB‐436‐RR cells were also markedly decreased by THOC5 knockdown (Figure 5E,F).

Furthermore, THOC5 depletion also significantly decreased the mRNA expression level of NANOG and SOX2 in 7–12 polysome fractions without affecting their total mRNA expression level (Figure 5G,H). To determine whether THOC5 mediates the nuclear export of NANOG and SOX2 transcripts in TNBC cells, we performed RNA‐FISH assays and analyzed the subcellular distribution of NANOG and SOX2 mRNAs after THOC5 knockdown. The results show that THOC5 silence significantly restrained NANOG and SOX2 mRNA export from the nucleus to the cytoplasm (Figure 5I). Additionally, through RIP assays, we observed that THOC5 silence abolished the binding of THOC2 to NANOG or SOX2 mRNAs in radioresistant TNBC cells (Figure 5J). These results collectively suggest that THOC5 plays an essential role in maintaining the cancer stemness and is required for THOC2 interaction with pluripotency gene transcripts in TNBC cells.

### 2.6 THOC2 or THOC5 Knockdown Suppresses the Tumorigenicity of Radioresistant TNBC In Vivo

**Figure 6:**
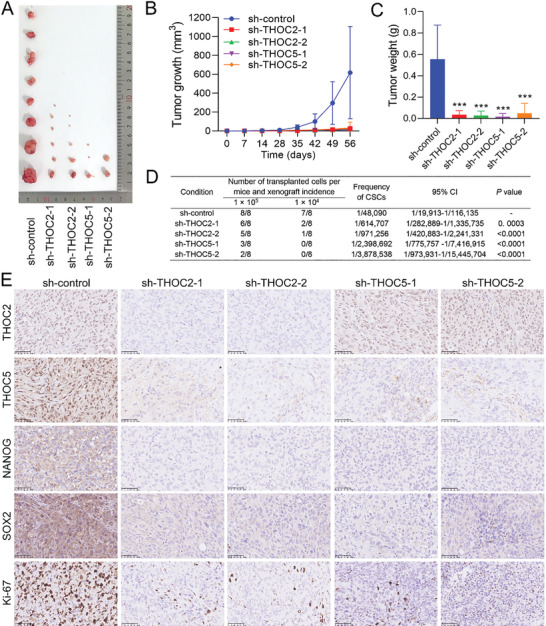
THOC2 or THOC5 knockdown suppresses the tumorigenicity of radioresistant TNBC in vivo. A) THOC2‐ or THOC5‐silenced MDA‐MB‐231‐RR cells were subcutaneously injected into the nude mice. The xenograft tumors derived from these MDA‐MB‐231‐RR cells were photographed and compared at the end of the experiment. B) Tumor growth was monitored every 4 days for 8 weeks, and the volume was recorded till the experiment ended. C) Tumor weight was measured and compared at the end of the experiment. D) Tumor incidence in mice transplanted with MDA‐MB‐231‐RR cells expressing sh‐control, sh‐THOC2, or sh‐THOC5 was shown and, a limiting dilution method was used to examine the frequency of CSCs in tumors. E) The protein level of THOC2, THOC5, NANOG, SOX2, and Ki‐67 in xenograft tumors was detected by IHC. Representative IHC images are shown at 200× magnification. ***P < 0.001 versus sh‐control group (*n* = 8).

To determine the effect of THOC2 or THOC5 depletion on the tumorigenic ability of radioresistant TNBC, we performed the in vivo experiment using a nude mouse xenograft model subcutaneously implanted with MDA‐MB‐231‐RR cells expressing sh‐control, sh‐THOC2, or sh‐THOC5. As depicted in **Figure** **6**A, THOC2 or THOC5 knockdown significantly decreased the number and size of tumors developed from MDA‐MB‐231‐RR cells and impaired their growth, as compared to the sh‐control group. Tumors from sh‐THOC2 or sh‐THOC5 groups grew slower than those in the sh‐control group and had much lower weight at the end of the experiment (Figure 6B,C). Analysis of the tumor incidence in vivo using a limiting dilution method demonstrates that the frequency of CSCs in tumors derived from THOC2‐ and THOC5‐depleted MDA‐MB‐231‐RR cells were much lower than that from sh‐control cells (Figure 6D). Further investigation by IHC analysis of the xenograft tumors shows that the intratumoral protein levels of THOC2 or THOC5 were markedly decreased by their specific shRNAs, and the protein expression of NANOG and SOX2 was concomitantly decreased (Figure 6E). In addition, a significant reduction of Ki‐67‐positive cells in the xenograft tumors was also found in the sh‐THOC2 and sh‐THOC5 groups (Figure 6E). These data indicate that THOC2 or THOC5 depletion can suppress the tumorigenicity and tumor growth of radioresistant TNBC in vivo.

### 2.7 THOC5 Knockdown Restores the Radiosensitivity of TNBC Cells

**Figure 7:**
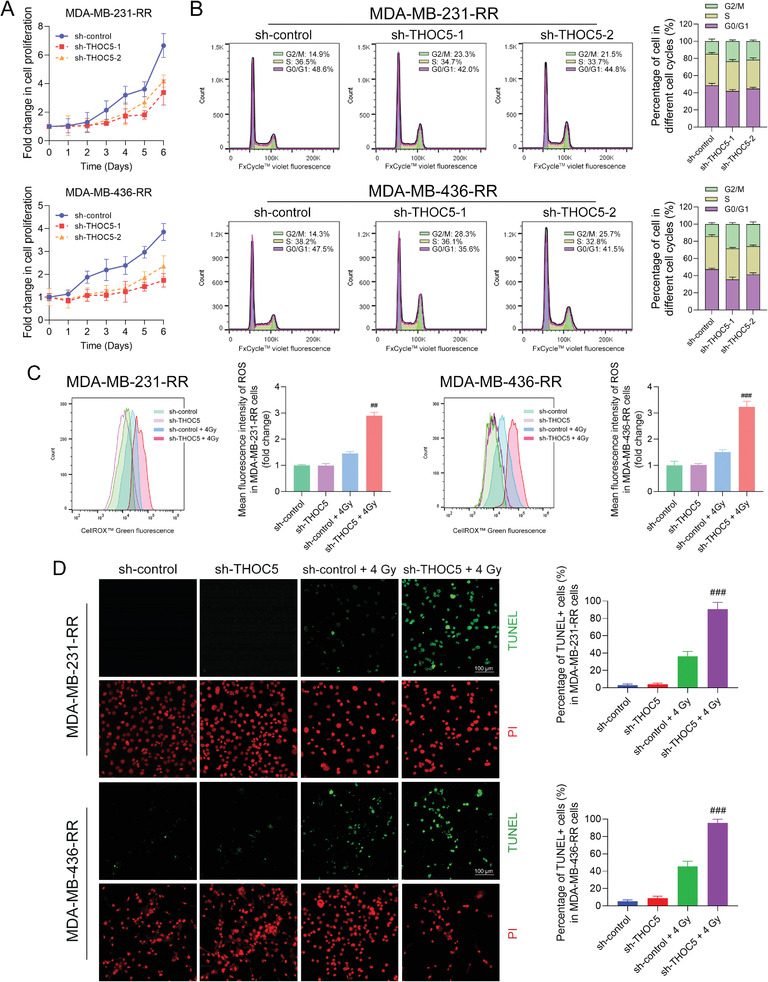
THOC5 knockdown restores the radiosensitivity of TNBC cells. THOC5 was silenced in MDA‐MB‐231‐RR and 436‐RR cells using a lentiviral system with two different shRNAs. A) The proliferation rate of cells was determined using the proliferation assay. B) The cell cycle was analyzed using flow cytometry. THOC5‐depleted MDA‐MB‐231‐RR and 436‐RR cells were treated with 0 or 4 Gy IR. C) The intracellular level of ROS was detected 24 h after IR using the flow cytometry. The mean fluorescence intensity of ROS was measured and compared. D) The cell apoptosis was evaluated 24 h after IR using the TUNEL assay. Representative images for the TUNEL assay were obtained at 100× magnification. Green fluorescence represents DNA double‐strand breaks, while red fluorescence represents the nuclei. The percentage of TUNEL^+^ cells was calculated and compared. ^##^ *P* < 0.01 and ^###^ *P* < 0.001 versus sh‐control + IR group (*n* = 3).

Consistent with the in vivo results, the cell proliferation assay shows that THOC5 silence impaired the proliferation of MDA‐MB‐231‐RR and 436‐RR cells (**Figure** **7**A). Further results from the cell cycle analysis show that THOC5 silence blocked the cell cycle progression of radioresistant TNBC cells, as evidenced by the cell cycle arrest in the G2/M phase, a critical checkpoint for damaged DNA (Figure 7B).

Next, we questioned whether the THOC5 knockdown could restore the radiosensitivity of TNBC. Hence, we first evaluated the effect of THOC5 knockdown in the intracellular level of ROS before and after IR using flow cytometry. The results show that THOC5 silence did not interfere with the ROS production in the absence of IR whereas dramatically increased 4 Gy IR‐induced intracellular accumulation of ROS in MDA‐MB‐231‐RR and 436‐RR cells (Figure 7C). The TdT‐mediated dUTP nick end labeling (TUNEL) assay allows the in situ detection of DNA breaks during cell apoptosis, which is a suitable method to assess the cell damage caused by IR. Through TUNEL assay, we observed that THOC5 silence combined with 4 Gy IR significantly increased DNA breaks, as shown by the increased TUNEL^+^ cells in radioresistant TNBC cell lines (Figure 7D). There is no markable difference in the percentage of TUNEL^+^ cells between the sh‐control and sh‐THOC5 groups in the absence of IR (Figure 7D). These results suggest that THOC5 depletion can reverse TNBC radioresistance. Additionally, THOC5 knockdown also increased the sensitivity of radioresistant TNBC cells to cisplatin and doxorubicin (Figure S4B, Supporting Information).

### 2.8 THOC2 Promotes Stemness and Radioresistance of TNBC Cells in a THOC5‐Dependent Manner

**Figure 8:**
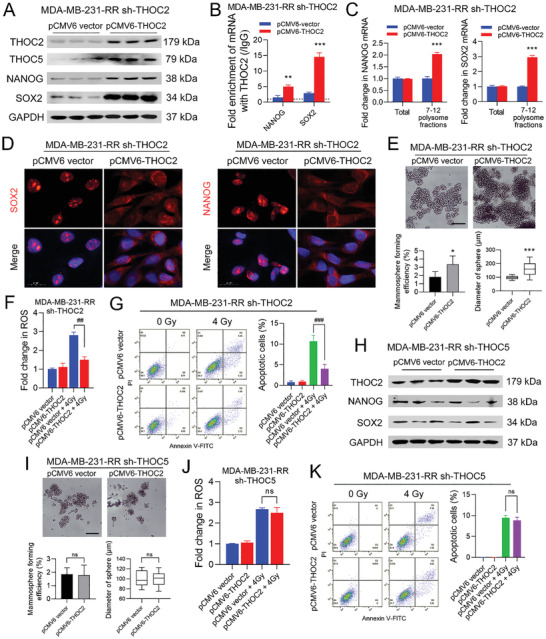
THOC2 promotes stemness and radioresistance of TNBC cells in a THOC5‐dependent manner. THOC2 was rescued in THOC2‐silenced MDA‐MB‐231‐RR cells by the transfection of a THOC2‐overexpressing *pCMV6* plasmid. A) The protein expression of THOC2, THOC5, NANOG, and SOX2 was detected by WB. GAPDH was used as the loading control. B) The enrichment of NANOG and SOX2 mRNAs against THOC2 and IgG antibodies in TNBC cell lines was analyzed by RIP analysis. C) The relative expression of polysome‐associated NANOG and SOX2 mRNAs (fractions 7–12) was detected by qRT‐PCR. D) The intracellular distribution of NANOG and SOX2 mRNAs was evaluated by RNA‐FISH. Representative images are shown at 630× magnification. Red represents target mRNA, while blue represents the nuclei. E) The stemness of cells was evaluated by mammosphere formation assay. The formation efficiency and sphere volume were calculated, and representative images are shown at 100× magnification. THOC2‐silenced MDA‐MB‐231‐RR cells were transfected with a THOC2‐overexpressing *pCMV6* plasmid and then treated with 0 or 4 Gy IR. F) The intracellular ROS level was detected 24 h after IR. G) The cell apoptosis was analyzed 24 h after IR using flow cytometry, and the percentage of apoptotic cells was calculated as the percentage of cells in Q2 and Q3. THOC2 was overexpressed in THOC5‐silenced MDA‐MB‐231‐RR cells by the transfection of a THOC2‐overexpressing *pCMV6* plasmid. H) The protein expression of THOC2, NANOG, and SOX2 was detected by WB. GAPDH was used as the loading control. I) The stemness of cells was evaluated by mammosphere formation assay. The formation efficiency and sphere volume were calculated, and representative images are shown at 100× magnification. THOC5‐silenced MDA‐MB‐231‐RR cells were transfected with a THOC2‐overexpressing *pCMV6* plasmid and then treated with 0 or 4 Gy IR. J) The intracellular ROS level was detected 24 h after IR. K) The cell apoptosis was analyzed 24 h after IR using flow cytometry, and the percentage of apoptotic cells was calculated as the percentage of cells in Q2 and Q3. ns, nonsignificant; *P < 0.05, **P < 0.01, and ***P < 0.001 versus vector group; ^##^ *P* < 0.01 and ^###^ *P* < 0.001 versus vector + IR group (*n* = 3).

To further validate the importance of THOC2 in maintaining TNBC stemness, THOC2 expression was rescued in THOC2‐depleted MDA‐MB‐231‐RR cells using a THOC2‐overexpressing plasmid (*pCMV6‐THOC2*) that is resistant to shRNA interference. The results from WB analysis show that the transfection of the *pCMV6‐THOC2* plasmid could restore the protein expression of THOC2, NANOG, and SOX2 in THOC2‐depleted MDA‐MB‐231‐RR cells (**Figure** **8**A). Further data from the RIP analysis demonstrate that the binding activity of THOC2 with NANOG and SOX2 mRNAs was recovered by the rescue of THOC2 (Figure 8B), and the amount of NANOG and SOX2 mRNAs in 7–12 polysome fractions in THOC2‐depleted MDA‐MB‐231‐RR cells was increased as well (Figure 8C). Consistently, SOX2 and NANOG transcripts were also predominantly localized in the cytoplasm after THOC2 rescue, as compared to the vector group (Figure 8D). The sphere‐forming ability of THOC2‐silenced MDA‐MB‐231‐RR cells was markedly reinstituted by THOC2 rescue (Figure 8E). To further validate the importance of THOC2 in sustaining TNBC radioresistance, we evaluated the sensitivity of THOC2‐depleted MDA‐MB‐231‐RR cells to IR‐induced oxidative stress and apoptosis after THOC2 rescue assay. As depicted in Figure 8F,G, THOC2 rescue by *pCMV6‐THOC2* plasmid significantly reduced 4 Gy IR‐induced intracellular ROS accumulation and cell apoptosis, as compared to the vector group.

To further validate the importance of THOC5 relative to THOC2, we upregulated THOC2 expression in THOC5‐depleted MDA‐MB‐231‐RR cells using the *pCMV6‐*THOC2 plasmid and assessed the effect on their stemness and radiosensitivity. Results from WB analysis show that the protein expression of NANOG and SOX2 was not altered by the THOC2 overexpression (Figure 8H). Consistently, THOC2 overexpression in THOC5‐depleted MDA‐MB‐231‐RR cells failed to reinstitute their mammosphere‐forming ability (Figure 8I). In addition, THOC2 upregulation in THOC5‐depleted MDA‐MB‐231‐RR cells did not reduce the intracellular ROS level and the percentage of apoptotic cells caused by 4 Gy IR (Figure 8J,K). Together, these data suggest that THOC2 critically functions in maintaining the stemness and radiosensitivity of TNBC by regulating pluripotency gene transcripts in a THOC5‐dependent manner.

## 3 Discussion

**Figure 9:**
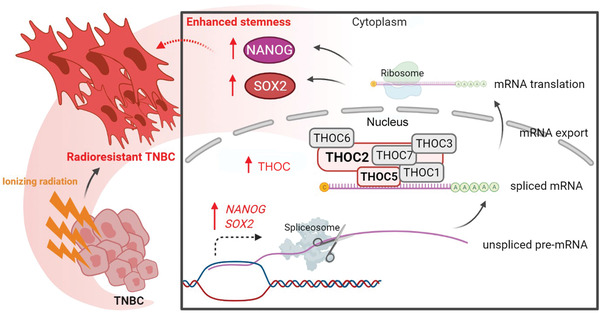
The schematic of the THOC‐mediated stemness enhancement and radioresistance in TNBC. The export of mRNA from the nucleus to the cytoplasm is a key step in protein synthesis, which is essential for all living eukaryotic cells. The THOC is a key component in the formation of cotranscription that messenger ribonucleoparticles can be transported into the cytoplasm for translation. THOC2 and THOC5, as the backbone protein and specific adaptor for the THOC, respectively, play a vital role in maintaining the protein expression of pluripotent transcription factors and the switching of embryonic stem cell differentiation and proliferation. In this study, we found that radioresistant TNBC can employ this mechanism via upregulating the protein expression of THOC2 and THOC5 to promote the THOC‐mediated spliced mRNA efflux, increase the translation and protein synthesis of NANOG and SOX2, and enhance the stem‐like properties of TNBC cells. The upregulation of NANOG and SOX2 gene expression also provides a prerequisite for this mechanism. The enhanced stemness thus confers TNBC cells a survival advantage upon RT‐induced oxidative stress injury and apoptosis. (This figure was created with Biorender.com)

The treatment option for TNBC patients is still very limited.^[^ ^25^ ^]^ Our previous findings showed that radioresistant TNBC cells display a cross‐resistance to cytotoxic agents,^[^ ^20^ ^]^ suggesting that exploring novel strategies to combat TNBC therapeutic resistance is of great significance. In the present study, we demonstrate several important novel findings: i) THOC2 and THOC5 are upregulated in radioresistant TNBC cells and associated with a worse prognosis in TNBC patients; ii) THOC2 regulates TNBC stemness by controlling the nuclear export of NANOG and SOX2 transcripts in a THOC5‐dependent manner; iii) THOC2 or THOC5 knockdown can disrupt TNBC stemness in vitro and in vivo and increase the radiosensitivity. An illustration of the proposed mechanism is shown in **Figure** **9**.

TNBC is further associated with the presence of CSCs compared with the luminal subtypes.^[^ ^26^ ^]^ These poorly differentiated cells with redundant signaling networks of stemness orchestrate the natural aggressiveness of TNBC, including distant metastasis and recurrence.^[^ ^21^ ^]^ The mesenchymal‐like tumor is a vital subtype of TNBC, characterized by a gene expression profile favorable to CSC maintenance, and has the worst outcome.^[^ ^27^ ^]^ The CD44^+^CD24^−/low^ CSCs are consistently enriched in this subtype.^[^ ^28^ ^]^ Most mesenchymal‐like MDA‐MB‐231 and 436 cells are marked with a CD44^+^CD24^−/low^ expression signature. Furthermore, ALDH^+^ CSCs were also identified from TNBC. The ALDH^+^CD44^+^CD24^−/low^ expression signature marks a more purified CSC subpopulation in TNBC.^[^ ^11^ ^]^ In addition, Liu et al.^[^ ^29^ ^]^ identified a population of CD133^+^ tumor cells from TNBC, with the capability to mimic embryonic vasculogenic networks and organize intratumoral vasculogenesis. These studies indicate that CSCs are heterogeneous and should be identified by a combination of markers. In this study, we not only characterized the expression profiles of CD44, CD24, CD133, and ALDH1A3 but also detected CSC subpopulations with ALDH^+^, CD133^+^, and CD44^+^CD24^−/low^ expression signature in radioresistant TNBC cell lines. Whilst the expression difference of these markers varies among different cell lines, the data collectively suggest that the CSCs were more enriched in radioresistant TNBC cells. Along with the data from mammosphere evaluation, these results depict an enhancement of stemness in radioresistant TNBC cells.

Previous studies have demonstrated that traditional therapies can enhance stem‐like properties in TNBC through inducing EMT,^[^ ^30^ ^]^ hypoxia,^[^ ^31^ ^]^ metabolic reprogramming,^[^ ^32^ ^]^ and stromal remodeling ^[^ ^14^ ^]^ that can promote the conversion of non‐CSCs to CSCs. Moreover, the hyperactivated self‐renewal signalings, such as Wnt/*β*‐catenin, Hedgehog, and Notch pathways, give CSCs a survival advantage upon the treatment‐induced DNA damage.^[^ ^33^ ^]^ Consequently, conventional treatments usually only kill fast‐dividing cancer cells but are ineffective against quiescent CSCs, which would increase the proportion of CSCs in the residual tumor and cause hidden risks for recurrence.^[^ ^21^ ^]^ The upregulation of core pluripotency factors, such as OCT4,^[^ ^34^ ^]^ SOX2,^[^ ^35^ ^]^ and NANOG,^[^ ^36^ ^]^ is often found in residual disease and exerts an unfavorable impact on the survival of patients with TNBC. These transcriptional factors are critical in maintaining the self‐renewal capacity and pluripotency of stem cells and have been considered valuable CSC markers. It was shown that the increase of OCT4,^[^ ^37^ ^]^ SOX2,^[^ ^38^ ^]^ and NANOG ^[^ ^39^ ^]^ could enhance the stemness and determine the malignant phenotypes in a wide range of cancers. Consistently, in the current study, we found that the expression of OCT4, SOX2, and NANOG was increased in radioresistant TNBC cells, suggesting that RT can enhance the stemness of TNBC by promoting the expression of core pluripotency master regulators.

The multisubunit THOC interacts with a couple of other proteins, such as ALYREF, UAP56, and CIP29, in an ATP‐dependent manner to form the TREX complex responsible for the coupling of transcription to mRNAs in a splicing‐dependent manner. This is essential for the effective export of polyadenylated RNAs and spliced mRNAs.^[^ ^16^ ^]^ Our data show that both THOC2 and THOC5, as well as their binding activities to pluripotency mRNAs, were upregulated in radioresistant TNBC cells, suggesting that they may play a role in maintaining the stemness of TNBC by coupling the pluripotency mRNAs. Silencing THOC2 or THOC5 disrupted the protein expression of NANOG and SOX2 but failed to affect their total mRNA expression, indicating that they were required for pluripotency protein level in TNBC and that the THOC regulates pluripotency gene at the posttranscriptional level. Further analysis of polysome association, which shows the translational efficiency for a given transcript, provides comparable evidence that THOC2 or THOC5 depletion post‐transcriptionally blocked the expression of pluripotency genes. To test our hypothesis that THOC2 and THOC5 control the releasing of pluripotency mRNAs from nuclear speckle domains in TNBC, we performed RNA‐FISH assay following THOC2 or THOC5 depletion using the probes that are designated to localize the specific mRNAs and confirmed that they are essential in mediating the nuclear export of NANOG and SOX*2* transcripts.

Notably, the mRNA expression of OCT4, NANOG, and SOX2 was also significantly upregulated in radioresistant TNBC cells, and this may be associated with the activation of the JAK2/STAT3 signaling. Studies from other groups have shown that the JAK2/STAT3 signaling is overactivated in radioresistant TNBC cells and closely associated with the mesenchymal‐like subtype of TNBC.^[^ ^27^, ^40^ ^]^ Also, it has been well documented that *SOX2* is a critical downstream gene for STAT3,^[^ ^41^ ^]^ and forced expression of constitutively active STAT3 can increase the transcription activity of *NANOG*, *SOX2*, and *OCT4* in TNBC cells, as demonstrated by the luciferase reporter assay.^[^ ^42^ ^]^ These studies suggest that the JAK2/STAT3 pathway may be an important signaling that triggers the mRNA expression of the pluripotency transcription factors in TNBC, which deserves further investigation. Following this potential regulatory mechanism, upregulated TOHC2 and THOC5 play a significant role in facilitating their mRNA export and translation, suggesting that targeting the THOC is a promising approach to eradicating TNBC stemness.

It is well established that THOC2 physically interacts with THOC5 and is a shared subunit for different THOCs as a scaffold to interact with nucleic acids directly.^[^ ^43^ ^]^ Since THOC2 silence can lead to THOC5 disappearance and reduce the population of CSCs in TNBC cells, we hypothesized that THOC2 might be a determinant factor in regulating the stemness of TNBC. The observation that the protein expression of THOC5, NANOG, and SOX2 was recovered upon the rescue of THOC2 supports our hypothesis. Furthermore, in this study, the role of THOC5 in maintaining cancer stemness was also established. THOC2 overexpression in THOC5‐depleted radioresistant TNBC cells failed to increase the protein expression of NANOG and SOX2 and rebuild the sphere‐forming ability, suggesting that the role of THOC2 in mediating the enhanced stem‐like properties of TNBC requires the functions of THOC5. To date, the knowledge about the role of the THOC in cancer stemness and therapeutic resistance is very limited. We demonstrate for the first time that THOC2 and THOC5 knockdown could compromise the tumorigenic capacity of TNBC cells in vitro and in vivo by disrupting the expression of pluripotent transcription factors and restore the sensitivity of radioresistant TNBC cells to IR. More importantly, we show that THOC2 and THOC5 were upregulated in human TNBC tissues and significantly associated with a worse survival rate in TNBC patients, highlighting the therapeutic value and targetable advantage of the THOC in cancer. Finding out inhibitors that can target the THOC might be of great significance for developing novel targeted drugs to combat TNBC via eradicating CSCs.

## 4 Conclusion

Targeting the CSC subpopulation is one of the most important strategies for treating malignant cancers, and eradicating intratumoral CSCs may particularly obtain a better outcome in TNBC patients. Given this, our study reveals that the THOC‐mediated transnuclear export of pluripotency transcripts plays a critical role in promoting cancer stemness and is a novel mechanism contributing to TNBC radioresistance. These findings propose THOC2 and THOC5 as novel therapeutic targets for TNBC by eliminating the stem‐like properties of the tumor, and provide molecular insight and rationale to develop the THOC2 or THOC5 inhibitors as targeted treatments for the recurrent TNBC.

## 5 Experimental Section

### Cell Culture

Thehuman TNBC cell lines (MDA‐MB‐231, 436, and 468) were obtained from the American Type Culture Collection (VA, USA). MDA‐MB‐231, 436, and 468 cells were cultured in the Iscove's modified Dulbecco medium (Gibco, NY, USA), RPMI‐1640 medium (Gibco), and Dulbecco's modified Eagle medium (DMEM) (Gibco), respectively, supplemented with 10% fetal bovine serum (FBS, Gibco) and 1% penicillin‐streptomycin solution (Gibco). Cells were maintained at 37 °C with humid air and 5% CO_2_ in an incubator. Radioresistant TNBC cell lines (MDA‐MB‐231‐RR, 436‐RR, and 468‐RR) were developed and verified as previously described.^[^ ^20^ ^]^ These TNBC cells are much more resistant to IR, as well as several chemotherapeutic drugs, than their parental cells and showed remarkedly radioresistant phenotype within 5–8 passages after establishment,^[^ ^20^ ^]^ whereas no significant radioresistance was observed after eight passages. Therefore, in this study, radioresistant TNBC cells were only used within five passages after establishment.

### Cell Proliferation Assay

Cells were seeded in the 96‐well plate with a density of 1 × 10^3^ per well and cultured for 6 days. Cell proliferation was detected by the Cell Counting Kit‐8 (CCK8) assay (Abcam, MA, USA). Briefly, CCK‐8 solution (10 µL) was added to each well and incubated with cells for 4 h at 37 °C in the dark. The absorbance of each well was then measured at 460 nm using the Multiskan FC microplate photometer (Thermo Scientific, CA, USA). Cell proliferation rate is presented as the fold change of absorbance.

### Colony Formation Assay

The exponentially growing cells were plated in 6‐well plates at the density of 5 × 10^2^ to 2 × 10^3^ per well and were cultured for 9–14 days. The colonies were then stained with 0.5% crystal violet (Solarbio, Beijing, China) for 15 min at room temperature, and the number of colonies (>50 cells) was counted and photographed.

### Mammosphere Formation Assay

Single cells were seeded into the ultralow attachment 24‐well plate (Corning, NY, USA) at a density of 2 × 10^3^ per well with serum‐free DMEM/F‐12 medium (Gibco) supplemented with 1 × B27 (Gibco), 0.4% bovine serum albumin (Solarbio), 0.5 µg mL^−1^ hydrocortisone (Solarbio), 4 µg mL^−1^ heparin (Solarbio), 4 µg mL^−1^ insulin (Solarbio), 20 ng mL^−1^ EGF (Solarbio), and 20 ng mL^−1^ bFGF (Solarbio). Cells were cultured for 5–7 days with 500 µL fresh medium added every 3 days, and spheres (> 50 µm) were then counted and photographed using a light field microscope equipped with a phase‐contrast module (Leica, Wetzlar, Germany). Mammosphere formation efficiency (MFE) was calculated as follows: MFE (%) = (the number of mammospheres identified per well/the number of cells seeded per well) × 100%. For the limiting dilution analysis of the mammosphere formation, single cells were seeded into the Corning Costar ultralow attachment 96‐well plate at the density of 1–2 × 10^2^ per well. After 5–7 days, the percentage of wells without spheres was plotted against the number of cells per well, and the regression lines were generated accordingly.

### Lentiviral Transduction

The stable knockdown of THOC2 or THOC5 was performed with lentiviral particles (*pGFP‐C‐shLenti*) containing genes encoding THOC2‐ or THOC5‐targeted shRNAs (Locus ID 57187 and 8563, OriGene Technologies, MD, USA). The vector was used as the scramble control (OriGene Technologies). The tansduction of lentiviral particles was performed with cells in a medium containing 8 µg mL^−1^ polybrene. After 18 h, the transduction efficiency was verified by flow cytometry. Transduced cells were cultured in the lentiviral particle‐free medium for another 72 h, and then 1 µg mL^−1^ puromycin (Solarbio) was used to select clones with stable shRNA expression. Both qRT‐PCR and WB analysis were used to confirm the knockdown of THOC2 and THOC5.

### Plasmid Transfection

THOC2 expression rescue was performed with a *pCMV6‐THOC2* plasmid containing a THOC2 sequence resistant to the specific shRNAs used in this study. The plasmid was designed and assembled by OriGene Technologies. The transfection was performed with the plasmid–lipid complex using the Lipofectamine 3000 (Thermo Scientific, CA, USA) according to the protocol. After 72 h, the transfected cells were subject to qRT‐PCR to confirm the expression of THOC2 and collected for the following assay.

### Western Blotting (WB)

The protein samples from TNBC cell lines were extracted using RIPA lysis buffer (Beyotime, Shanghai, China) supplemented with 1 × Halt protease and phosphatase inhibitor cocktail (Thermo Scientific). The equal amount of protein (10‐20 µg) was separated on a 10% SDS‐PAGE gel, transferred to a 0.45 µm immobilon PVDF membrane (Millipore, CA, USA), and then blocked with 5% BSA. After incubation with primary antibodies (Abs) at 4 °C overnight, protein bands were further incubated with HRP‐conjugated secondary antibody. Unless otherwise specified, Abs were diluted 1:1000. The protein lanes were illuminated by an enhanced chemiluminescence substrate (Beyotime) and exposed in the ImageQuant LAS 4000 system (GE Healthcare, IL, USA). The GAPDH was used as an internal reference. The rabbit primary polyclonal antibodies against ALDH1A3 (ab129815), THOC2 (ab129485), and cleaved caspase‐9 (ab2324) were purchased from Abcam (MA, USA). The rabbit monoclonal antibodies against CD44 (ab189524), CD24 (ab179821), CD133 (ab216323), THOC5 (ab137051), OCT4 (ab181557), NANOG (ab214549), SOX2 (ab92494), cleaved caspase‐3 (ab32042), cleaved PARP1 (ab32064), and GAPDH (ab181602, dilution ratio 1:2000) were purchased from Abcam. The antirabbit secondary antibody (7074) was purchased from Cell Signaling Technology (MA, USA).

### Quantitative Real‐Time PCR (qRT‐PCR)

The MiniBEST Universal RNA Extraction Kit (Takara Bio, Kyoto, Japan) was used to extract total RNA from cells. Reverse transcription was performed using 1 µg total RNA and PrimeScript RT reagent Kit (Takara Bio), and then the qRT‐PCR reaction was performed with 2 µL cDNA and SYBR Premix Ex Taq II (Takara Bio) on the 7500 Real‐Time PCR System (Applied Biosystems, CA, USA). Primers for human OCT4 (qHsaCED0038334), NANOG (qHsaCED0043394), SOX2 (qHsaCED0036871), and GAPDH (qHsaCED0038674) were purchased from BioRad (CA, USA). GAPDH was used as an internal reference. The relative gene expression was calculated using the 2^−ΔΔCT^ method.

### Reactive Oxygen Species (ROS) Detection

Adherent cells were seeded overnight and treated with 4 Gy IR in the culture medium. After 24 h, CellROX Green Reagent Kit (Thermo Scientific) was used to detect intracellular ROS levels. Briefly, CellROX Green reagent was added to the cells with the final concentration of 5 × 10^−6^ m and 800 × 10^−9^ m for assays of microplate reader and flow cytometry, respectively, and incubated at 37 °C for 30 min in the dark. The stained cells were washed in buffer, and the fluorescence intensity was measured using the Infinite M200 PRO microplate reader (Tecan, Männedorf, Switzerland) at excitation/emission = 485/520 nm or flow cytometer (LSR Fortessa X‐20, BD Bioscience, CA, USA) at the 450/50 bandpass filter.

### Flow Cytometry Analysis

Cell apoptosis analysis was performed after 24 h using the Annexin V‐FITC Apoptosis Detection Kit (Abcam) on the flow cytometer at the 530/30 bandpass filter for annexin V‐FITC and the 610/20 bandpass filter for propidium iodide (PI). The cell cycle was determined using the FxCycle Violet Stain Kit (Thermo Scientific) according to the protocol at the 450/50 bandpass filter. The ALDEFLUOR Kit (STEMCELL Technologies, MA, USA) was used to analyze the percentage of ALDH^+^ cells in the same flow cytometer. The FITC mouse antihuman CD44 (555478, BD Bioscience, 1:100) and PE mouse antihuman CD24 (555428, BD Bioscience, 1:100) Abs were used to analyze the percentage of CD44^+^CD24^−/low^ cells. The FITC mouse antihuman CD133 (567033, BD Bioscience, 1:100) was used to determine the percentage of CD133^+^ cells.

### Polysome Preparation

Cells were incubated with 100 µg mL^−1^ cycloheximide (CST) in the medium at 37 °C for 10 min and were lysed in polysome lysis buffer containing 1% Triton X‐100 (CST). The polysome lysate was centrifugated and added to the top of the sucrose solutions of ten different densities (bottom to top: 50–5%) filled in an ultracentrifuge tube. The polysomes were size fractionated by ultracentrifuge at 36 000 rpm for 2 h at 4 °C using the SW 41 Ti Swinging‐Bucket Rotor (Beckman Coulter, IN, USA). A total of twelve fractions was collected from top to bottom of the sucrose gradient using the density gradient fractionator (Brandel, MD, USA). The total RNA of each fraction was isolated and subject to qRT‐PCR analysis.

### RNA Immunoprecipitation (RIP)

RIP assay was performed using the Magna RIP RNA‐Binding Protein Immunoprecipitation Kit (MilliporeSigma, MA, USA). Cells were lysed in the RIP lysis buffer with a 1 × protease inhibitor cocktail and RNase inhibitor. Magnetic bead‐bound antibodies and IgG were produced by incubating the antibodies and IgG with magnetic beads for 30 min at room temperature. Cell lysates were then incubated with appropriate antibodies or IgG at 4 °C overnight in the RIP immunoprecipitation buffer with rotating. Magnetic beads were collected and incubated with the proteinase K buffer at 55 °C for 30 min. The aqueous phase was incubated with the salt solution I, II, precipitate enhancer, and absolute ethanol at −80 °C overnight and was centrifuged at 140 00 rpm for 15 min at 4 °C to collect the RNA for qRT‐PCR analysis.

### RNA Fluorescence In Situ Hybridization (FISH)

RNA‐FISH assay was performed with the ViewRNA ISH Cell Assay Kit (ThermoFisher Scientific). After fixation by 4% formaldehyde, cells on poly‐l‐lysine coated coverslips were rehydrated, permeabilized, and digested with the protease solution. The predesigned specific probes for NANOG (Assay ID: VA6‐11054‐VC) and SOX2 (Assay ID: VA6‐11765‐VC) mRNAs were purchased from the ThermoFisher Scientific and prepared in the probe set diluent QF. Cells were incubated with the probes at 40 °C for 3 h, the PreAmplifier Mix solution at 40 °C for 30 min, and the Label Probe Mix solution at 40 °C for 30 min. The 4,6‐diamidino‐2‐phenylindole (DAPI) solution was used to stain the nuclei, and the images were photographed immediately using an FV300/FV500 confocal microscope (Olympus, Tokyo, Japan).

### TdT‐Mediated dUTP Nick end Labeling (TUNEL) Assay

TUNEL assay was performed using the TUNEL Assay Kit‐FITC (Abcam). After IR, cells were fixed in ice‐cold 70% ethanol and washed with Wash Buffer. DNA labeling solution was prepared with Reaction Buffer, TdT Enzyme, FITC‐dUTP, and ddH_2_O according to the kit specification. Cells were incubated with the labeling solution for 60 min at 37 °C and then PI/RNase Staining Buffer in the dark for 30 min at room temperature. Stained cells were photographed immediately using the IX73 inverted fluorescence microscope (Olympus).

### Mouse Xenograft Experiments

Female athymic (nu/nu) BALB/c mice (5 week old, 18–20 g) were provided by Guangdong Medical Laboratory Animal Center (Guangzhou, China). The mice were housed in a specific pathogen‐free (SPF) animal facility at the Laboratory Animal Research Center of Sun Yat‐sen University (Guangzhou, China), and they were allowed free access to SPF‐grade food and water. All procedures involving mice were approved by the Animal Care and Use Committee of Sun Yat‐sen University (Approval No. IACUC‐2020‐B0813). After 1 week adaptation, the mice were randomly divided into ten groups with eight mice per group: 1) sh‐control‐a, 2) sh‐THOC2‐1a, 3) sh‐THOC2‐2a, 4) sh‐THOC5‐1a, 5) sh‐THOC5‐2a, 6) sh‐control‐b, 7) sh‐THOC2‐1b, 8) sh‐THOC2‐2b, 9) sh‐THOC5‐1b, 10) sh‐THOC5‐2b. Pre‐prepared MDA‐MB‐231‐RR cells in the logarithmic growth phase were collected in 1 × PBS. The 1 × 10^5^ and 1 × 10^4^ cells in 0.1 mL 1 × PBS were injected into the right flank of the mouse in the a and b sequence, respectively, to perform the limiting dilution assay. The tumor size was measured using a caliper every 4 days, and the formula (length × width^2^ × 0.5) was used to calculate the volume. The frequency of CSCs in tumors from different groups was analyzed using the L‐Calc software (STEMCELL Technologies). Two months after injection, all mice were euthanized, and tumors were separated into fresh frozen stored at −80 °C and formalin‐fixed for paraffin embedding.

### Tissue Microarray (TMA) and Immunohistochemistry (IHC)

Human TMA slides (BRC1601) were purchased from Superbiotek (Shanghai, China). Xenograft tumor tissues were embedded in paraffin and cut into 5 × 10^−6^ m sections mounted on slides. These tissue sections, once melted onto slides at 60 °C, were first deparaffinized with xylene and dehydrated with graded ethanol. All sections were retrieved in 0.01 m citrate buffer for 15 min at 95 °C and blocked with goat serum. After incubation with primary antibodies at 4 °C overnight, the slides were incubated with the secondary antibody for 1 h at room temperature. The staining of protein was displayed by adding the 3,3‐diaminobenzidine liquid substrate (Agilent, CA, USA). The staining intensity was scored using a light microscope (Leica) as follows: 0 (negative, <25%), 1 (weak, 25–50%), 2 (moderate, 50–75%), 3 (strong, >75%). Rabbit primary mAbs against Ki‐67 (ab16667), SOX2 (ab92494), and NANOG (ab214549) were purchased from Abcam. Rabbit primary pAb against THOC2 (ab129485) was purchased from Abcam. Rabbit primary pAb against THOC5 was obtained from Sangon Biotech (D153425, Shanghai, China).

### Bioinformation Analysis

Analysis of the clinical proteomic tumor analysis consortium (CPTAC) datasets was performed in the UALCAN platform (http://ualcan.path.uab.edu).^[^ ^44^ ^]^ Protein expressions were *Z*‐score transformed, and the Wilcoxon rank‐sum test was used to compare the protein expression among different groups in the box‐and‐whisker plot (Figure 2B). In addition, a total of 126 TNBC cases and 65 BC cases, with intratumoral proteomics data and clinical annotations, were obtained from the work of Liu et al. ^[^ ^23^ ^a]^ and Tang et al.^[^ ^23^ ^b]^, respectively. The cutoff value for high and low expression of a protein was optimized by the X‐Tile,^[^ ^45^ ^]^ and the Kaplan–Meier survival curve was generated accordingly and compared by the log‐rank in the Prism 9 (GraphPad, CA, USA) (Figure 2F,G).

### Statistical Analysis

Data were analyzed using Prism 9 and shown as the mean ± standard deviation. Two‐tailed Student's *t*‐test and one‐way ANOVA with Tukey's test were used to compare the difference between two groups and among three or more groups, respectively. For the analysis of Figure 1A, two‐way ANOVA with Šidák correction was used in duplicates. The comparison of the IHC score between adjacent normal and TNBC tissues in TMA was performed using paired *t*‐test (Figure 2D). Multivariate analysis was performed with the Cox proportional hazard regression model using the SPSS Statistics 25 software platform (IBM, NY, USA) to evaluate the predictive value of prognostic factors for OS (Figure 2E). Sample sizes (*n*) and probability (*P*) values are indicated in the figure legends. *P* < 0.05 was considered statistically significant.

## Conflict of Interest

The authors declare no conflict of interest.

## Author Contribution

Y.L. and X.B. assisted in concept and design; X.B. and Y.L. assisted in data collection, analysis, and interpretation; X.B., J.N., J.B., and Y.L. assisted in writing, reviewing, and/or revising manuscripts; Y.L., P.G., S.W., J.N., J.B., and X.D. assisted in administrative, technical, or material support.

## Supporting information

Supporting Information

## Data Availability

The data that support the findings of this study are available from the corresponding author upon reasonable request.
