# Supplementary material for: THOC2 and THOC5 Regulate Stemness and Radioresistance in Triple‐Negative Breast Cancer
Source: Adv Sci (Weinh). 2021 Oct 27;8(24):2102658. doi: 10.1002/advs.202102658 (PMC8693071; doi:10.1002/advs.202102658)
Supplement: Supplementary file 1 — Supporting Information [file ADVS-8-2102658-s001.pdf]

© 2021 Wiley-VCH GmbH

ADVANCED SCIENCE
Open Access

Supporting Information

for *Adv. Sci.*, DOI: 10.1002/advs.202102658

THOC2 and THOC5 Regulate Stemness and Radioresistance in Triple-Negative Breast Cancer

Xupeng Bai, Jie Ni, Julia Beretov, Shanping Wang, Xingli Dong, Peter Graham, and *Yong Li**

Supporting Information

THOC2 and THOC5 Regulate Stemness and Radioresistance in Triple-Negative Breast Cancer

*Xupeng Bai, Jie Ni, Julia Beretov, Shanping Wang, Xingli Dong, Peter Graham, Yong Li**

1. Supporting Experimental Methods

1.1 Primers for qRT-PCR

The primers used for the qRT-PCR analysis of THO components were purchased from BioRad: human THOC1 (qHsaCID0017129), human THOC2 (qHsaCID0007436), human THOC3 (qHsaCED0037925), human THOC5 (qHsaCID0010424), human THOC6 (qHsaCED0044398), and human THOC7 (qHsaCID0008771).

1.2 Chemosensitivity assay

The powder of cisplatin and doxorubicin was purchased from Solarbio (Beijing, China). The cisplatin or doxorubicin store solution was prepared in dimethyl sulfoxide (DMSO) at the concentration of 20 mM and 2 mM, respectively, and was used by a dilution rate of 1: 1000 in the culture medium. Treatment with 0.1% DMSO was used as the control. Before testing the chemosensitivity, cells were seeded in the 96-well plate with a density of 5×10^3 per well and allowed to adhere overnight. After treatment with drugs for 48 h, cell viability was determined using the Cell Counting Kit-8 (CCK8) assay (Abcam, MA, USA) and measured at 460 nm using the Multiskan™ FC microplate photometer (Thermo Scientific, CA, USA). Cell viability (%) = absorbance (drug-treated group) / absorbance (control group) × 100%.

1.3 Probes for RNA FISH

The pre-designed specific probes for OCT4 (Assay ID: VA6-17268-VC) mRNAs were purchased from ThermoFisher Scientific.

1.4 Bioinformatic analysis

Analysis of the Cancer Genome Atlas (TCGA) datasets was performed in the UALCAN platform (http://ualcan.path.uab.edu). For the Molecular Taxonomy of Breast Cancer International Consortium (METABRIC) dataset, a total of 2509 BC cases with annotation and RNA-sequencing data were obtained through the cBioPortal (https://www.cbioportal.org). Thereinto, 298 TNBC cases were included according to the 'negative' in the fields of HER2 status, ER status, and PR status. The X-Tile software was used to optimize the cut-off value for high and low expression of a gene, and the Kaplan-Meier survival curve was generated

accordingly and compared by the log-rank test.

1.5 Statistical analysis

Pearson's $\chi2$ test was used to analyze the correlation of THOC2 and THOC5 expression with different clinicopathological variables from tissue microarray (TMA).

2. Supporting Figures

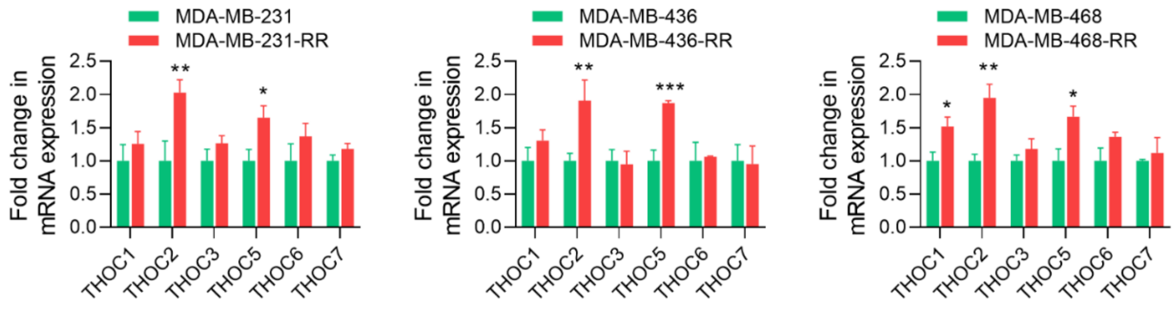

Figure S1. The mRNA expression of THOC2 and THOC5 is up-regulated in radioresistant TNBC. The relative mRNA expression of THOC1, THOC2, THOC3, THOC5, THOC6, and THOC7 in TNBC cell lines was detected by qRT-PCR. $^{*}P < 0.05$, $^{**}P < 0.01$, and $^{***}P < 0.001$ vs. parental cells ($n = 3$).

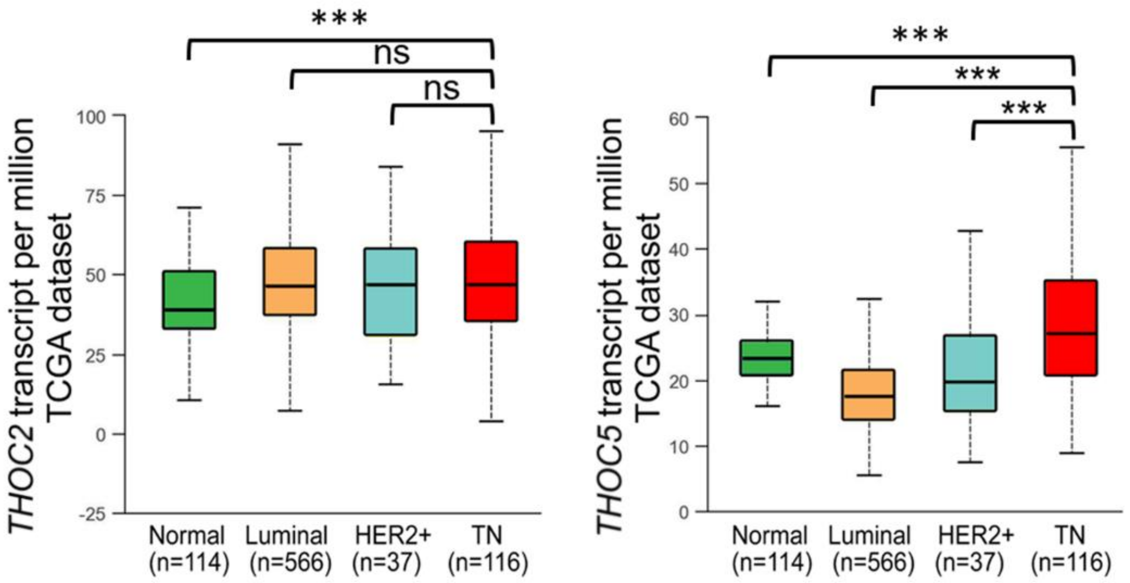

Figure S2. The expression of *THOC2* and *THOC5* is up-regulated in TNBC tumors. The box-and-whisker plots were generated using the UALCAN to describe *THOC2* and *THOC5* gene expression differences among human normal breast tissues and different BC subtypes. The gene copy number was obtained from the TCGA-BRCA dataset. $^{***}P < 0.001$ vs. other tissues; ns, non-significant.

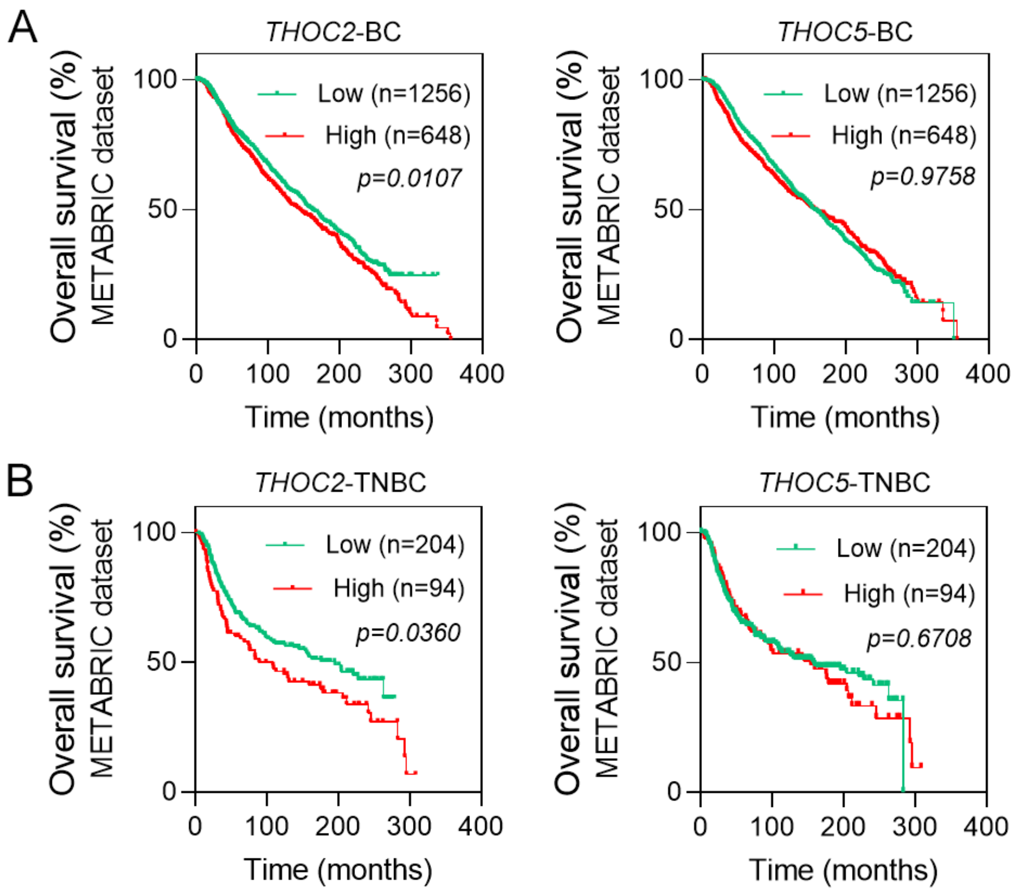

Figure S3. The intratumoral mRNA expression of THOC2 is associated with a worse overall survival (OS) in breast cancer (BC) patients. **(A)** The association of *THOC2* and *THOC5* expression with OS was analyzed in BC cohorts from the METABRIC dataset. **(B)** The association of *THOC2* and *THOC5* expression with OS was analyzed in TNBC cohorts from the METABRIC dataset.

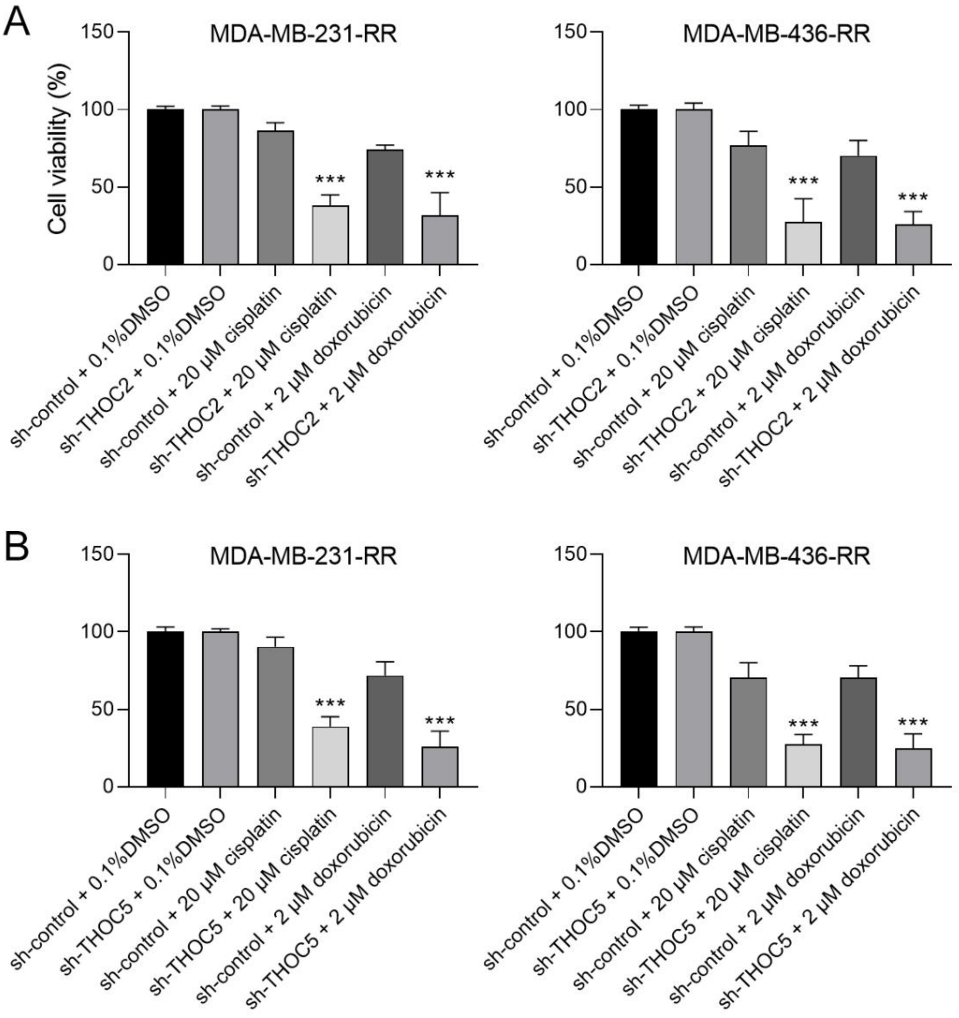

Figure S4. THOC2 or THOC5 knockdown sensitizes radioresistant TNBC to chemotherapy. (A) THOC2 was knocked down in MDA-MB-231-RR and -436-RR cells. These cells were then treated with 20 μM cisplatin or 2 μM doxorubicin for 48 h. **(B)** THOC5 was knocked down in MDA-MB-231-RR and -436-RR cells. These cells were then treated with 20 μM cisplatin or 2 μM doxorubicin for 48 h. Cell viability was determined with the CCK8 assay. $^{###}P < 0.001$ vs. sh-control group (n = 5).

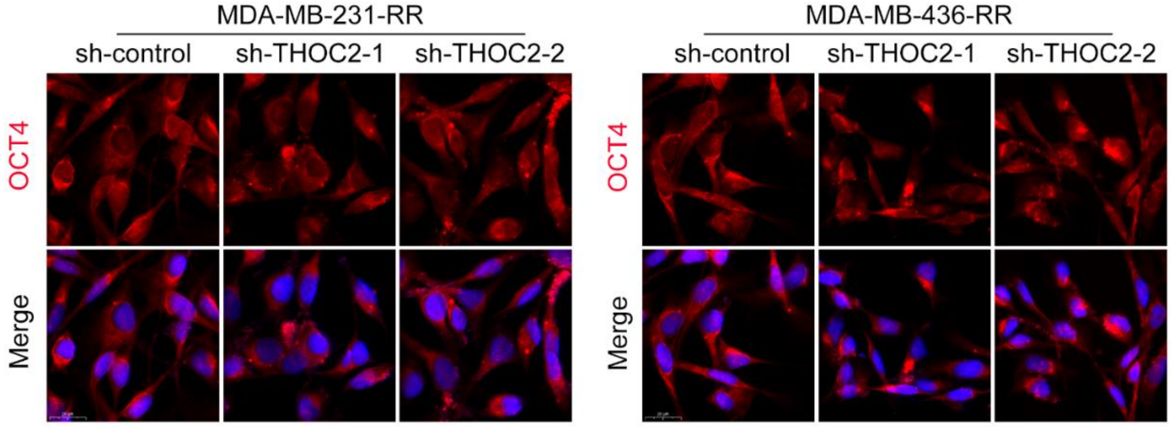

Figure S5. THOC2 knockdown did not affect the nuclear transport of OCT4 transcripts in radioresistant TNBC. The intracellular distribution of OCT4 mRNAs was evaluated by RNA-FISH. Representative images were shown at 630× magnification. Red represents target mRNA, while blue represents the nuclei.

3. Supporting Tables

Table S1. Correlation analysis of THOC2 expression with clinicopathological variables from TMA.

Variables	Total (n = 43)	THOC2 expression		*P*-value
		Low (IHC score 1) (n = 24)	High (IHC score 3) (n = 19)	
**Age (years)**				
< 45	10	3 (30.0%)	7 (70.0%)	0.061
≥ 45	33	21 (63.6%)	12 (36.4%)	
**Tumor size**				
< 2 cm	13	4 (23.1%)	9 (76.9%)	**0.029**
≥ 2 cm	30	20 (66.7%)	10 (33.3%)	
**Lymph node invasion**				
Positive	26	11 (42.3%)	15 (57.7%)	**0.027**
Negative	17	13 (76.5%)	4 (23.5%)	
**Histological grade**				
G1-G2	21	13 (61.9%)	8 (38.1%)	0.432
G3	22	11 (50.0%)	11 (50.0%)	
**Stage**				
0-I	8	6 (75.0%)	2 (25.0%)	0.226
IIA or higher	35	18 (51.4%)	17 (48.6%)	
**THOC5 expression**				
Low (IHC score 1)	35	24 (68.6%)	11 (31.4%)	**<0.001**
High (IHC score 3)	8	0 (0.0%)	8 (100%)	

Table S2. Correlation analysis of THOC5 expression with clinicopathological variables from TMA.

Variables	Total (n = 50)	THOC5 expression		*P*-value
		Low (IHC score 1) (n = 30)	High (IHC score 3) (n = 20)	
**Age (years)**				
< 45	13	7 (53.8%)	6 (46.2%)	0.599
≥ 45	37	23 (62.2%)	14 (37.8%)	
**Tumor size**				
< 2 cm	9	5 (55.6%)	4 (44.4%)	0.764
≥ 2 cm	41	25 (61.0%)	16 (39.0%)	
**Lymph node invasion**				
Positive	26	15 (57.7%)	11 (42.3%)	0.729
Negative	24	15 (62.5%)	9 (37.5%)	
**Histological grade**				
G1-G2	24	15 (62.5%)	9 (37.5%)	0.729
G3	26	15 (57.7%)	11 (42.3%)	
**Stage**				
0-I	10	7 (70.0%%)	3 (30.0%)	0.541
IIA or higher	40	23 (57.5%)	17 (42.5%)	
**THOC2 expression**				
Low (IHC score 1)	41	29 (70.7%)	12 (29.3%)	**<0.001**
High (IHC score 3)	9	1 (11.1%)	8 (88.9%)	
